# Clinical evidence of an interferon–glucocorticoid therapeutic synergy in COVID-19

**DOI:** 10.1038/s41392-021-00496-5

**Published:** 2021-03-03

**Authors:** Yingying Lu, Feng Liu, Gangling Tong, Feng Qiu, Pinhong Song, Xiaolin Wang, Xiafei Zou, Deyun Wan, Miao Cui, Yunsheng Xu, Zhihua Zheng, Peng Hong

**Affiliations:** 1 Department of Biomedical Science, Shenzhen Research Institute, City University of Hong Kong, Kowloon Tong, Hong Kong, China; 2 Department of Nephrology, Center of Nephrology and Urology, Sun Yat-sen University Seventh Hospital, Shenzhen, Guangdong China; 3 Department of Infectious Diseases, Suizhou Zengdu Hospital, Suizhou, Hubei China; 4 Department of Oncology, Peking University Shenzhen Hospital, Shenzhen, Guangdong China; 5 Intensive Care Unit, Suizhou Zengdu Hospital, Suizhou, Hubei China; 6 Department of Respiratory Medicine, Suizhou Zengdu Hospital, Suizhou, Hubei China; 7 Department of Pathology, Mount Sinai St. Luke’s Roosevelt Hospital Center, New York, NY USA; 8 Department of Dermatology, Sun Yat-sen University Seventh Hospital, Shenzhen, Guangdong China; 9 Division of Research and Development, US Department of Veterans Affairs New York Harbor Healthcare System, Brooklyn, NY USA; 10 Department of Cell Biology, State University of New York Downstate Health Sciences University, Brooklyn, NY USA

**Keywords:** Immunotherapy, Respiratory tract diseases, Infectious diseases

## Abstract

Synthetic glucocorticoid dexamethasone is the first trial-proven drug that reduces COVID-19 mortality by suppressing immune system. In contrast, interferons are a crucial component of host antiviral immunity and can be directly suppressed by glucocorticoids. To investigate whether therapeutic interferons can compensate glucocorticoids-induced loss of antiviral immunity, we retrospectively analyzed a cohort of 387 PCR-confirmed COVID-19 patients with quasi-random exposure to interferons and conditional exposure to glucocorticoids. Among patients receiving glucocorticoids, early interferon therapy was associated with earlier hospital discharge (adjusted HR 1.68, 95% CI 1.19–2.37) and symptom relief (adjusted HR 1.48, 95% CI 1.06–2.08), while these associations were insignificant among glucocorticoids nonusers. Early interferon therapy was also associated with lower prevalence of prolonged viral shedding (adjusted OR 0.24, 95% CI 0.10–0.57) only among glucocorticoids users. Additionally, these associations were glucocorticoid cumulative dose- and timing-dependent. These findings reveal potential therapeutic synergy between interferons and glucocorticoids in COVID-19 that warrants further investigation.

## Introduction

Recent reports from both bench and bedside suggest dysregulated activation of host immune response to severe acute respiratory syndrome coronavirus 2 (SARS-CoV-2) as a pivotal feature of severe novel corona virus disease 2019 (COVID-19), which prompts reconsideration of glucocorticoid (GC) therapy to restrain the hyperactivated immune system.^1–4^ Despite of their unrivaled anti-inflammatory efficacy, high-dose GC were associated with severe adverse effects and should be cautioned in treating COVID-19 patients.^5^ However, clinical efficacy of low to moderate-dose GC on COVID-19 has been controversial,^6–8^ until the recent announcement of preliminary results from the UK RECOVERY trial, which showed significant reduction of COVID-19 mortality by synthetic GC dexamethasone (DEX) in severe patients receiving invasive mechanical ventilation or oxygenation.^9^ Further clinical evidence showed that high-dose methylprednisolone, another synthetic GC, was associated with clinical benefits in COVID-19-associated cytokine storm syndrome (CSS)^10^ and systemic GC was associated with lower 28-day all-cause mortality in a prospective meta-analysis of 7 randomized clinical trials of critically ill patients with COVID-19.^11^ Nevertheless, the potent anti-inflammatory effects of GC also weaken innate antiviral immunity, leading to delayed viral clearance and adverse outcomes in various viral pneumonias.^12–14^ Therefore, whether GC are sufficiently beneficial to non-severe COVID-19 patients remains an open question.

Interferon (IFN) signaling is a crucial component of human antiviral immunity that restricts viral replication and spreading.^15^ GC has been reported to directly suppress IFN responses in a chronic obstructive pulmonary disease (COPD) mouse model, leading to impaired lung virus control which can be reversed by therapeutic IFN.^16^ Interestingly, a signature immune response upon SARS-CoV-2 infection is low IFN levels in peripheral blood and lungs of severe COVID-19 patients, which may be part of the immune evasion mechanisms of SARS-CoV-2.^17,18^ Potentially by targeting this mechanism, IFN therapies using IFN-β1a alone or IFN-β1b in combination with lopinavir-ritonavir have both shown promising results in treating COVID-19 patients.^19,20^ A number of trials involving IFN as mono or combination therapy for COVID-19 are ongoing, which may provide a more definitive verdict for its efficacy in COVID-19. However, IFN may play a more damaging role by disrupting lung epithelial repair at the later stage of disease,^21,22^ and it is thus crucial to pre-determine the extent of viral infection and the stage of pathogenesis, which would ensure the right timing of IFN therapy.

Based on the above-mentioned evidences,^16,19,20^ we hypothesize that IFN therapy may synergize with GC in treating COVID-19. To test this hypothesis, we identified a hospital in Hubei, China with high prevalence of IFN and GC therapies among COVID-19 patients and studied a cohort of 387 PCR-confirmed COVID-19 patients. Analyses using Cox proportional hazards and logistic regression models revealed that concomitant exposure to GC and early IFN therapy led to early hospital discharge, symptom relief and viral clearance than GC alone, while early IFN therapy without GC use was not associated with early COVID-19 recovery than standard care. This therapeutic synergy is dependent on the timing of GC administration and effective on GC doses used by the RECOVERY trial.

## Results

### Patient characteristics

A total of 406 patient records have been reviewed and 3 were excluded due to missing more than 5 days of treatment information before admission or during hospitalization. Another 16 were excluded from the study due to undergoing no antiviral therapies or extended hospital stay for conditions unrelated to COVID-19 (2 cases of liver diseases, 2 cases of lung tumors and 1 case of brain cancer). Among the 387 abstracted records, 118 (30.5%) received both GC and early IFN therapy, which has been defined empirically as initiation of IFN therapy within 5 days after admission and was shown to be associated with reduced mortality of COVID-19,^23^ 95 (24.5%) received GC alone, 87 (22.5%) received early IFN therapy alone, and 87 (22.5%) received neither drugs. The empirical criterion for early IFN therapy was partly based on the observation that all severe patients in this cohort developed ARDS or required ICU admission later than 5 days after admission. The median time from symptom onset to admission is 6 days (Table 1), which would qualify IFN administered around 6 to 11 days after symptom onset as early therapy. This time window was justified by data from the same region showing that median time from disease onset to ARDS or ICU admission are 12 days.^24^ The overall in-hospital mortality of this cohort is 3.4%, which is in line with the average COVID-19 mortality in Hubei province excluding Wuhan city during January to March 2020.^25^ Mortality was observed in 11 (11.6%) patients with GC alone and 2 (1.7%) patients with both GC and early IFN therapy (Supplementary Fig. 1), which was in agreement with our previous findings of a negative association between early IFN therapy and COVID-19 mortality.^23^ Because GC were given to all intensive care patients suffering respiratory distress, it was not feasible to assess the IFN-GC synergy regarding COVID-19 mortality based on this cohort. Instead, we focused on the recovery time of survivors based on length of hospital stay (LOS), time from admission to symptom relief, and incidence of prolonged viral shedding (PVS). Among the entire cohort, the median LOS is 20 (interquartile range [IQR], 15–26) days, and the median time from admission to symptom relief is 12 (IQR, 9–16) days. These values were comparable to those reported based on data from the same area.^26^ The overall prevalence of PVS, which was defined as at least one positive SARS-CoV-2 PCR test after symptom relief, is 14.7%.

**Table 1:** Characteristics of survivors by treatment group

	No./total No. (%)						
	With GC therapy		P* (IFN + GC vs GC)	No GC therapy		P* (IFN vs no IFN/GC)	P* (All 4 groups)
	Early IFN (*n* = 116)	No early IFN (*n* = 84)		Early IFN (*n* = 87)	No early IFN (*n* = 87)		
*Demographic characteristics*							
Female sex	49 (42.2)	45 (53.6)	0.118	38 (43.7)	41 (47.1)	0.761	0.420
Age, years, median (IQR)	51 (42.5–58.5)	51 (42.5–62.5)	0.750	48 (34–54)	47 (35–56)	0.989	0.016
>60	26 (22.4)	23 (27.4)	0.506	9 (10.3)	17 (19.5)	0.136	0.039
Hypertension	30 (25.9)	19 (22.6)	0.622	13 (14.9)	11 (12.6)	0.826	0.064
Diabetes	14 (12.1)	8 (9.5)	0.651	1 (1.1)	3 (3.4)	0.621	0.008
High-risk exposure	64 (55.2)	55 (65.5)	0.148	59 (67.8)	43 (49.4)	0.021	0.041
*Clinical features (Within 24* *h of admission)*							
Symptom onset to hospital admission, days, median (IQR) [No.]	6 (4–8)	6 (4–9)	0.862	6 (4–9) [86]	6 (4–8) [85]	0.572	0.901
>7 days	34 (29.3)	25 (29.8)	1.00	27 (31)	22 (25.3)	0.500	0.853
Abnormal CT findings	116 (100)	84 (100)	/	86 (98.9)	85 (97.7)	1.00	0.241
Respiratory rate (RR) > 22/min	21 (18.1)	16 (19)	0.856	9 (10.3)	9 (10.3)	1.00	0.174
O_2_ saturation (O_2_ST), %							
>93	73 (62.9)	54 (64.3)	0.329	84 (96.6)	75 (86.2)	0.048	<0.001
90–93	34 (29.3)	19 (22.6)		3 (3.4)	11 (12.6)		
<90	9 (7.8)	11 (13.1)		0 (0)	1 (1.1)		
Fever	98 (84.5)	75 (89.3)	0.404	71 (81.6)	71 (81.6)	1.00	0.472
Symptom count^a^							
1	23 (19.8)	27 (32.1)	0.019	26 (29.9)	31 (35.6)	0.672	0.091
2	65 (56)	49 (58.3)		47 (54)	45 (51.7)		
3	23 (19.8)	8 (9.5)		13 (14.9)	9 (10.3)		
4 or more	5 (4.3)	0 (0)		1 (1.1)	2 (2.3)		
Lymphopenia (<1.1×10^9^/L)	62 (62.6) [99]	28 (54.9) [51]	0.383	22 (31) [71]	23 (35.4) [65]	0.715	<0.001
Eosinopenia (<0.02×10^9^/L)	80 (81.6) [98]	34 (68) [50]	0.067	36 (52.2) [69]	31 (49.2) [63]	0.862	<0.001
Severity category at admission^b^							
Moderate	73 (62.9)	54 (64.3)	0.408	84 (96.6)	75 (86.2)	0.015	<0.001
Severe	41 (35.3)	26 (40.0)		3 (3.4)	12 (13.8)		
Critical	2 (1.7)	4 (4.8)		0 (0)	0 (0)		
*Treatments*							
Time from admission to first dose of GC, days, median (IQR)	2 (1–4)	4 (2–6)	<0.001	/	/	/	/
Within 5 days	110 (94.8)	60 (71.4)	<0.001	/	/	/	/
Time from symptom onset to first dose of GC, days, median (IQR)	9 (6.5–11)	11 (8–14)	0.001	/	/	/	/
Duration of GC treatment, days, median (IQR)	4 (3–7)	4 (3–6)	0.319	/	/	/	/
Peak daily GC dose (methylprednisolone-equivalent, mg)							
40 mg or lower	52 (44.8)	39 (46.4)	0.478	/	/	/	/
80 mg	62 (53.4)	45 (53.6)		/	/		
160 mg or higher	2 (1.7)	0 (0)		/	/		
Cumulative dose of GC (methylprednisolone-equivalent, mg), median (IQR)	220 (120–400)	220 (120–360)	0.393	/	/	/	/
320 or less	80 (69)	61 (72.6)	0.639	/	/	/	/
800 or less	113 (97.4)	83 (98.8)	0.641	/	/	/	/
Time from admission to first dose of IFN, days, median (IQR) [No.]	2 (1–3) [116]	10.5 (7–16) [16]	<0.001	2 (1–2) [87]	7.5 (7–8) [4]	<0.001	<0.001
Duration of IFN therapy, days, median (IQR) [No.]	11 (8–14) [116]	9 (5.5–12) [16]	0.094	9 (7–12) [87]	8 (6.5–10) [4]	0.404	0.024
Lopinavir/ritonavir	39 (33.6)	49 (58.3)	0.001	32 (36.8)	43 (49.4)	0.126	0.002
Umifenovir	72 (62.1)	37 (44)	0.014	52 (59.8)	42 (48.3)	0.171	0.034
Oseltamivir	9 (7.8)	33 (39.3)	<0.001	14 (16.1)	18 (20.7)	0.558	<0.001
Human immune globulins	11 (9.5)	13 (15.5)	0.270	3 (3.4)	2 (2.3)	1.00	0.004
Antibiotics	106 (91.4)	67 (79.8)	0.021	65 (74.7)	66 (75.9)	1.00	0.007
*Overall*							
Length of hospital stay, days, median (IQR)	19 (15.5–26.5)	25.5 (22–31.5)	<0.001	18 (15–25)	17 (12.5–22)	0.092	<0.001
Time from admission to symptom relief, days, median (IQR)	12 (9–16)	14.5 (11–20)	<0.001	11 (9–14)	10 (7–14.5)	0.101	<0.001
Time from symptom onset to hospital discharge, days, median (IQR) [No.]	27 (22–34)	33 (25.5–39)	<0.001	25 (22–31) [86]	24 (19.5–30) [85]	0.171	<0.001
Retrospective severity category^b^							
Moderate	69 (59.5)	60 (71.4)	0.203	84 (96.6)	79 (90.8)	0.295	<0.001
Severe	27 (23.3)	15 (17.9)		2 (2.3)	5 (5.7)		
Critical	20 (17.2)	9 (10.7)		1 (1.1)	3 (3.4)		
Mechanical ventilation	6 (5.2)	5 (6)	1.00	0 (0)	2 (2.3)	0.497	0.111
Prolonged viral shedding after symptom relief	13 (11.2)	33 (39.3)	<0.001	21 (24.1)	12 (13.8)	0.121	<0.001

*GC* glucocorticoids, *IFN* interferons, *IQR* inter quartile range, *CT* computed tomography

^*^P-values were calculated by Fisher’s exact test or Chi-square test for categorical variables, Mann–Whitney or Kruskal–Wallis tests for continuous variables

^a^Symptoms include fever, cough, throat discomfort, shortness of breath or chest discomfort, muscle weakness or pain, diarrhea, and headache

^b^Severity was retrospectively determined according to the national guideline: moderate disease has fever, respiratory symptoms and positive CT findings; severe disease has additionally RR > = 30, O_2_ST < = 93%, or partial pressure of oxygen (PaO_2_)/fraction of inspired oxygen(FiO_2_) <=300 mmHg; critical disease has additionally shock, respiratory distress requiring mechanical ventilation, or ICU admission

Due to the non-randomized assignment of GC to more severe patients, survivors were stratified by their exposure to GC before subgrouping based on exposure to early IFN therapy (Supplementary Fig. 2). Since IFN was used quasi-randomly with other antiviral therapies during the early breakout, demographic and early clinical characteristics of survivors were comparable between early IFN users and nonusers (Table 1). Notable exceptions were lower prevalence of lopinavir/ritonavir (LPV/r, Kaletra) and oseltamivir (Tamiflu) use and higher prevalence of umifenovir (Arbidol) use among patients with early IFN therapy than those without.

### Treatment descriptions

For GC treatments, only cumulative doses of at least 40 mg methylprednisolone were recorded. GC were mainly given in the early stage of hospitalization with cumulative doses equal to or less than 150 mg DEX (equivalent to 800 mg methylprednisolone) which was successfully used in a recent trial of acute respiratory distress syndrome (ARDS) (Supplementary Fig. 3),^27^ and more than 2/3 patients received cumulative doses equal to or less than 60 mg DEX (equivalent to 320 mg methylprednisolone) (Table 1), the maximum dose of the RECOVERY trial.^9^ Comparing early IFN and no early IFN groups, GC therapy was given at comparable dosages over comparable durations, resulting in similar cumulative doses (Table 1). Regarding IFN therapy, both GC users and nonusers in the early IFN group initiated IFN therapy of 5 million units in 2 mL sterile water via a nebulizer twice a day at a median time of 2 days after admission, while GC users have received IFN for longer durations than nonusers (median, 11 vs 9 days). Among patients without early IFN therapy, the timing and duration of IFN therapy were not statistically different between GC users and nonusers. Of note, all documented therapies in this study except GC have been used consecutively for at least 3 days following guidelines.^28^

Sensitivity analyses based on Cox proportional hazards model for LOS suggested LPV/r, oseltamivir, antibiotics, O_2_ saturation, gender, age, hypertension, and diabetes to be included in multivariable analyses as confounders (Supplementary Fig. 4a, b).

### GC or IFN was not independently associated with COVID-19 recovery

Among survivors, cumulative incidence curves for hospital discharge and symptom relief showed slower recovery of GC users than nonusers (Supplementary Fig. 5a, b), while the prevalence of PVS was comparable between GC users and nonusers that ceased viral shedding before discharge or death (Supplementary Fig. 5c). After adjusting for confounders, the associations of GC therapy with hospital discharge and symptom relief were no longer significant (Supplementary Fig. 5d). In contrast, cumulative incidence curves indicated marginal association of early IFN with earlier discharge and symptom relief (Supplementary Fig. 6a, b). The prevalence of PVS was also lower among patients receiving early IFN (Supplementary Fig. 6c). These associations also became insignificant after adjusting for confounders (Supplementary Fig. 6d).

### Early IFN therapy was associated with earlier recovery in GC users

**Fig. 1:**
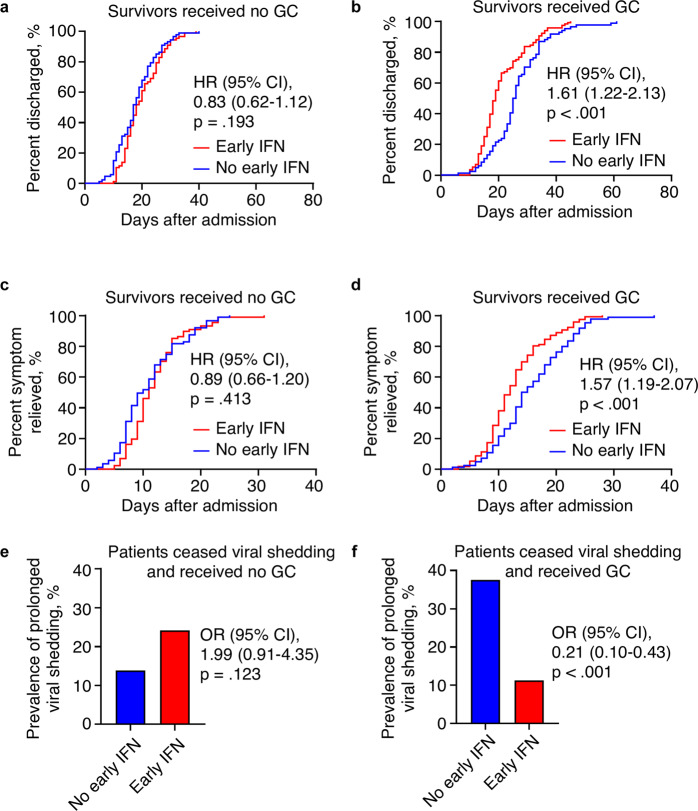
Association of early IFN therapy with early recovery among COVID-19 patients with GC therapy. **a**, **b** Cumulative incidence curves for hospital discharge among survivors without (**a**) or with GC (**b**). n = 87 for **a**, 116 (early IFN) and 84 (no early IFN) for **b**. **c**, **d** Cumulative incidence curves for symptom relief among survivors without (**c**) or with GC (**d**). n = 87 for **c**, 116 (early IFN) and 84 (no early IFN) for **d**. **e**, **f** Bar graph of PVS prevalence among patients ceased viral shedding before discharge without (**e**) or with GC (**f**). *n* = 87 for **e**, 116 (early IFN) and 88 (no early IFN) for **f**

After stratification by exposure to GC, cumulative incidence curves showed association of early IFN therapy with early hospital discharge among patients receiving GC (hazard ratio [HR] 1.61, 95% confidence intervals [CI] 1.22–2.13), which was not observed among patients without GC (HR 0.83, 95% CI 0.62–1.12) (Fig. 1a, b). Similarly, early IFN therapy was associated with early symptom relief only in GC users (HR, 95% CI: 1.57, 1.19–2.07 [with GC] vs 0.89, 0.66–1.20 [without GC]) (Fig. 1c, d). Moreover, early IFN therapy was associated with lower prevalence of PVS among GC users (odds ratio [OR] 0.21, 95% CI 0.10–0.43), while an insignificant but opposite trend was observed in nonusers (OR 1.99, 95% CI 0.91–4.35) (Fig. 1e, f). After adjusting for confounders, early IFN therapy was estimated to have HR (95% CI) of 1.68 (1.19–2.37) for hospital discharge and 1.48 (1.06–2.08) for symptom relief among GC users, which were reduced to 0.81 (0.59–1.12) and 0.87 (0.64–1.19) among nonusers, respectively (Table 2). Likewise, early IFN therapy had an adjusted OR (95% CI) of 0.24 (0.10–0.57) for PVS among GC users, which was increased to 2.05 (0.88–4.74) among nonusers (Table 2). Additionally, generalized log-gamma regression showed robust interaction between GC and early IFN therapy in estimating LOS and time to symptom relief values (*p* < 0.001 for both dependents). These data suggest potential synergy between GC and IFN in COVID-19.

**Table 2:** Model-adjusted risks of early interferon therapy

			Estimate (95% CI)			
Risk type	Outcome	Model	No GC (*n* = 174)	*P-*value	With GC (*n* = 200)	*P*-value
Hazard ratio	Hospital discharge	Cox proportional hazards^a^	0.81 (0.59–1.12)	0.197	1.68 (1.19–2.37)	0.003
Hazard ratio	Symptom relief	Cox proportional hazards^a^	0.87 (0.64–1.19)	0.377	1.48 (1.06–2.08)	0.023
Odds ratio	Prolonged viral shedding	Logistic regression^a^	2.05 (0.88–4.74)	0.096	0.24 (0.10–0.57)	0.001

*GC* glucocorticoids, *CI* confidence interval

^a^Model adjusted for gender, hypertension, diabetes, oxygen saturation at admission, symptom count at admission, lopinavir/ritonavir, oseltamivir, and umifenovir use. Cox models were fitted to all survivors, and logistic regression were fitted to patients ceased viral shedding before discharge or death

### Early IFN therapy could reduce GC adverse effects on COVID-19 recovery

**Fig. 2:**
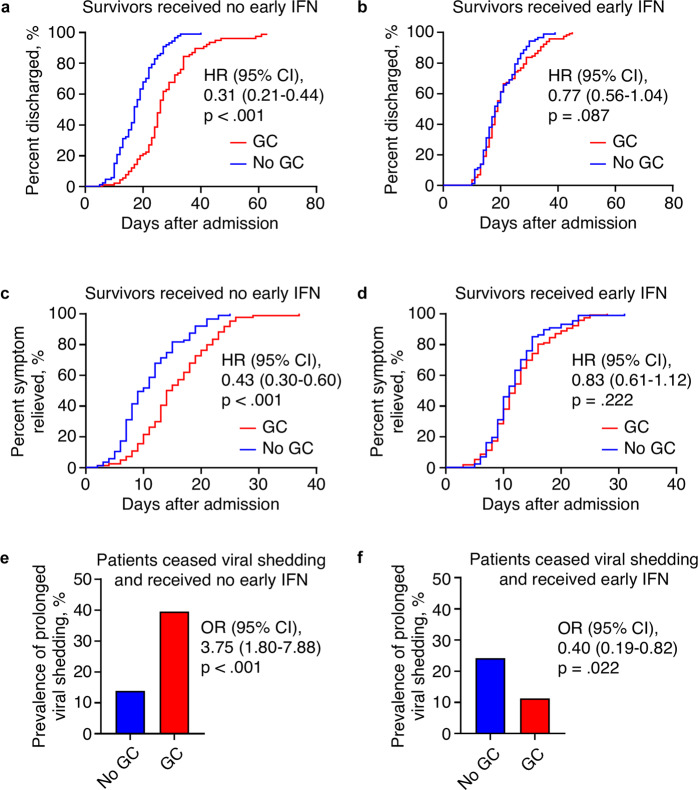
Association of early IFN therapy with reduced GC adverse effects on COVID-19 recovery. **a**, **b** Cumulative incidence curves for hospital discharge among survivors without (**a**) or with early IFN therapy (**b**). n = 84 (GC) and 87 (no GC) for **a**; 116 (GC) and 87 (no GC) for **b**. **c**, **d** Cumulative incidence curves for symptom relief among survivors without (**c**) or with early IFN therapy (**d**). *n* = 84 (GC) and 87 (no GC) for **c**; 116 (GC) and 87 (no GC) for **d**. **e**, **f** Bar graph of PVS prevalence among patients ceased viral shedding before discharge and without (**e**) or with early IFN therapy (**f**). *n* = 88 (GC) and 87 (no GC) for **e**; 116 (GC) and 87 (no GC) for **f**

Conversely, we evaluated the GC–IFN synergy from the perspective of GC. After stratification of survivors by exposure to early IFN therapy, GC were associated with delayed discharge and symptom relief that can be neutralized by early IFN therapy (Fig. 2a–d). Without early IFN therapy, GC users had prevalence of PVS 3 times higher than nonusers, while GC users showed lower prevalence of PVS than nonusers among those receiving early IFN therapy (Fig. 2e, f). After adjusting for confounders, GC were estimated to have HR (95% CI) of 0.56 (0.39–0.80) for hospital discharge and 0.65 (0.46–0.93) for symptom relief among survivors with no early IFN therapy, while among those receiving early IFN therapy the ratios increased to 1.13 (0.81–1.58) and 1.15 (0.82–1.60), respectively (Table 3). Likewise, GC were estimated to have an adjusted OR (95% CI) of 3.74 (1.64–8.53) for PVS in patients without early IFN therapy, which was reversed to 0.49 (0.20–1.21) in patients receiving early IFN therapy (Table 3). These findings suggest that the negative effects of GC on COVID-19 recovery may be neutralized by early IFN therapy and support the therapeutic synergy between GC and IFN. However, the observed GC negative effects may be confounded by the conditions that warrant GC therapy.

**Table 3:** Model-adjusted risks of GC therapy

			Estimate (95% CI)			
Risk type	Outcome	Model	No early IFN (*n* = 171)	*P*-value	With early IFN (*n* = 203)	*P-*value
Hazard ratio	Hospital discharge	Cox proportional hazards^a^	0.56 (0.39–0.80)	0.002	1.13 (0.81–1.58)	0.464
Hazard ratio	Symptom relief	Cox proportional hazards^a^	0.65 (0.46–0.93)	0.018	1.15 (0.82–1.60)	0.416
Odds ratio	Prolonged viral shedding	Logistic regression^a^	3.74 (1.64–8.53)	0.002	0.49 (0.20–1.21)	0.121

*GC* glucocorticoids, *CI* confidence interval

^a^Model adjusted for gender, hypertension, diabetes, oxygen saturation at admission, symptom count at admission, lopinavir/ritonavir, oseltamivir, and umifenovir use. Cox models were fitted to all survivors, and logistic regression were fitted to patients ceased viral shedding before discharge or death

### Early co-administration of GC and IFN led to stronger synergy

**Fig. 3:**
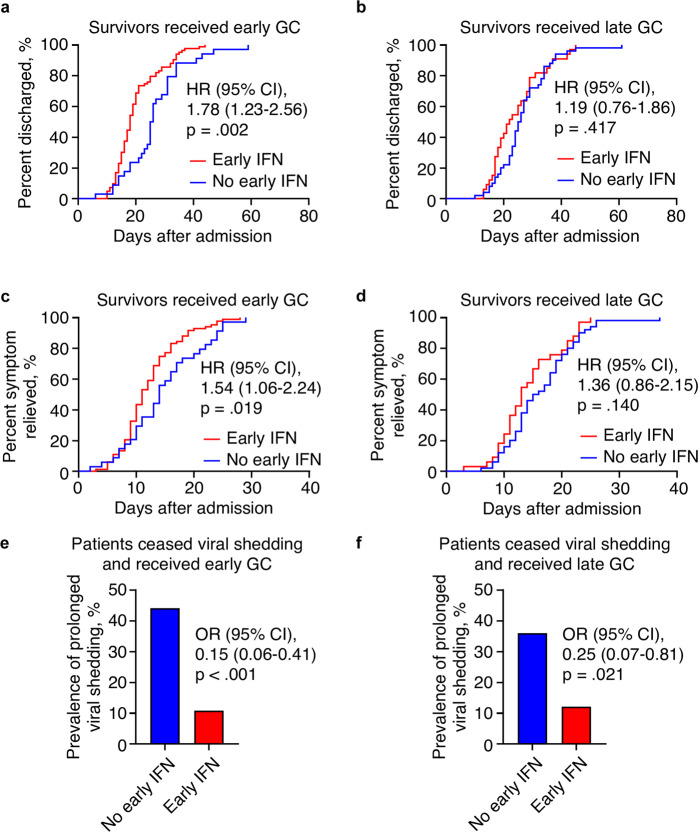
Association of early IFN therapy with early recovery from COVID-19 required early GC administration. **a**, **b** Cumulative incidence curves for hospital discharge among survivors with early (**a**) or late GC administration (**b**). *n* = 83 (early IFN) and 34 (no early IFN) for **a**, 33 (early IFN) and 50 (no early IFN) for **b**. **c**, **d** Cumulative incidence curves for symptom relief among survivors with early (**c**) or late GC administration (**d**). *n* = 83 (early IFN) and 34 (no early IFN) for **c**, 33 (early IFN) and 50 (no early IFN) for **d**. **e**, **f** Bar graph of PVS prevalence among patients ceased viral shedding before discharge and with early (**e**) or late GC administration (**f**). *n* = 83 (early IFN) and 38 (no early IFN) for **e**, 33 (early IFN) and 50 (no early IFN) for **f**

Recent evidence suggests COVID-19 immunopathogenesis as a multi-step process that closely correlated with disease severity and prognosis.^29^ To examine whether this GC–IFN synergy is timing-dependent, we defined early GC therapy as initiation of GC therapy within 5 days after admission, which overlapped with early IFN therapy, and stratified GC users according to this criterion. Analyses indicated that co-administration of GC and IFN at early stage was associated with shorter hospitalization than early GC users without IFN (HR 1.78, 95% CI 1.23–2.56), while early IFN therapy was not associated with hospital discharge in late GC users (HR 1.19, 95% CI 0.76–1.86) (Fig. 3a, b). Early administration of GC and IFN was also associated with early symptom relief than early GC users without IFN (HR 1.54, 95% CI 1.06–2.24), which was not statistically significant in late GC users (HR 1.36, 95% CI 0.86–2.15) (Fig. 3c, d). PVS prevalence was lower in patients with early IFN therapy among both early and late GC users, while early IFN therapy had an even lower OR among early GC users (OR [95% CI], 0.15 [0.06–0.41] vs 0.25 [0.07–0.81]) (Fig. 3e, f). These data suggest that the GC–IFN synergy is timing-dependent and administration of GC and IFN in the same window during early hospitalization was associated with the most benefits in recovery time.

### GC–IFN synergy was GC dose-dependent

**Fig. 4:**
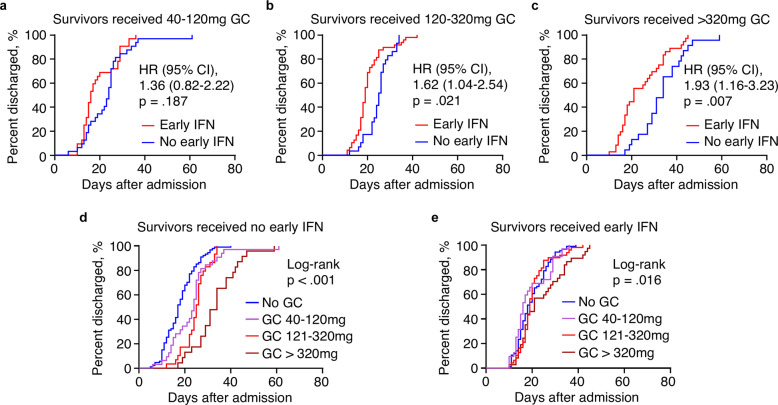
Association of early IFN therapy with early recovery from COVID-19 depended on GC cumulative dose. **a**–**c** Cumulative incidence curves for hospital discharge among survivors receiving 40–120 mg (**a**), 120–320 mg (**b**), or >320 mg methylprednisolone-equivalent doses of GC in total during hospitalization. *n* = 32 for **a**, 48 (early IFN) and 29 (no early IFN) for **b**, 36 (early IFN) and 23 (no early IFN) for **c**. **e**, **f** Cumulative incidence curves for hospital discharge among survivors without (**e**) or with early IFN therapy (**f**). *P*-values were calculated by comparing all 4 curves. *n* = 87 (no GC), 32 (GC 40–120 mg), 29 (GC 121–320 mg), and 23 (GC > 320 mg) for **e**, 87 (no GC), 32 (GC 40–120 mg), 48 (GC 121–320 mg), and 36 (GC > 320 mg) for **f**

Next, we evaluated whether GC cumulative dosage was correlated with GC–IFN synergistic effects on COVID-19 recovery, when IFN therapy was used in fixed dose and duration according to the guideline.^28^ We reasoned that true therapeutic synergy would be timing- and dose-dependent while synergy confounded by clinical indications of GC use would not. Thus, GC users were stratified by their cumulative methylprednisolone-equivalent doses into 3 groups: 40–120 mg, which showed benefit in an experimental study,^6^ 121–320 mg which represents doses used by RECOVERY trial,^9^ and >320 mg which represents higher doses effective against ARDS.^27^ Since the number of patients in each group was limited after stratification, only LOS was assessed due to its higher statistical power (Table 2). The analyses showed GC dose-dependent association of early IFN therapy with shorter hospitalization, with HR (95% CI) of 1.36 (0.82–2.22), 1.62 (1.04–2.54) and 1.93 (1.16–3.23) in 40–120 mg, 120–320 mg and >320 mg groups, respectively (Fig. 4a–c). Furthermore, analyses of LOS after stratifying the cohort by early IFN therapy showed that exposure of early IFN therapy reduced LOS of GC users at all doses (Fig. 4d, e). These findings suggest that the GC–IFN synergy is not only timing-relevant but also dose-dependent, which is significant at doses used by the RECOVERY trial.

## Discussion

More than 9 months into the COVID-19 pandemic, DEX remains the first and only randomized controlled trial-proven drug that reduces COVID-19 mortality,^9^ after conflicting results from recent large-scale trials seriously disputed the efficacy of Remdesivir.^30,31^ These findings, together with the recent success of DEX in treating ARDS, favor the perception that targeting CSS might be a more effective therapeutic approach than targeting viral replication to prevent fatal lung damage caused by the acute inflammation associated with severe COVID-19.^32^ However, we also have to acknowledge that DEX and other therapeutic GC are far from perfect drugs. Among the various adverse effects of GC, the suppression of antiviral immunity is particularly troublesome to viral pneumonias such as COVID-19, which may explain the potentially harmful effect of DEX in non-severe COVID-19.^9^ In this study, we analyzed a cohort of moderate to severe COVID-19 patients with high prevalence of GC and IFN exposures and identified an IFN–GC synergy that reduced GC-mediated suppression of antiviral immunity which adversely affected COVID-19 recovery. GC at doses used in the RECOVERY trial were not associated with delayed hospital discharge, symptom relief and viral clearance among patients receiving early IFN therapy, which was in sharp contrast with apparent GC-dependent delay of recovery observed in patients without early IFN therapy. This protective effect of early IFN therapy was observed regardless of timing and cumulative dosage of GC therapy. Mechanistically, we speculate that therapeutic IFN may compensate GC-dependent loss of IFN-dependent antiviral immunity and protect against the delay of viral clearance and disease recovery caused by GC therapy as observed in experimental viral infections after GC therapy.^16^ Additionally, IFN themselves have recently shown promising therapeutic potential against COVID-19 with molecular mechanisms involving both anti-viral replication and suppression of CSS.^17,18,23,33^ These evidences clearly warrant further research and randomized trials of IFN and GC as a combination therapy for COVID-19.

Our findings echoed the report of RECOVERY trial which concluded that dexamethasone reduced COVID-19 mortality only in those receiving mechanical ventilation or oxygen alone but not among those receiving no respiratory support.^9^ We reason that this discrepancy of therapeutic efficacy in severe or non-severe COVID-19 is the result of the dual-edge sword nature of GC therapy.^34^ On one hand, GCs suppress the life-threatening cytokine storm in the lung and prevent inflammation-induced tissue damage.^35^ On the other hand, GCs also impair the antiviral immunity which both delays viral clearance and increases the risk of secondary infections.^16^ GC inhibition of humoral response is a long-standing concern for its use in any infectious diseases, and this topic is still an ongoing debate in this era of COVID-19.^35,36^ Both animal studies and clinical observations showed that GC impair the immune response to viral infections and delay viral clearance.^13,14,16^ A recent observational study of COVID-19 patients with rheumatic diseases revealed regular GC use as the only risk factor of mortality among all the disease-modifying antirheumatic drugs, and GC were also associated with more frequent hospitalization.^37^ However, considering current guidelines only recommend GC as a temporary or last resort option for rheumatic disorders,^38^ those regular GC users may have more severe immunological disorders and thus were more susceptible to COVID-19 even without GC use. Nonetheless, both clinical and experimental evidence for the adverse effects of low dose GC, such as those used in the RECOVERY trial, on COVID-19 disease progress is still inconclusive. The RECOVERY trial used low dose of dexamethasone (6 mg/d) over a duration of 10 days,^9^ while the recent successful trial of dexamethasone in ARDS used high dose (20 mg/d) over a shorter period of 5 days followed by a withdraw period of 5 days.^27^ Previous experience during the SARS outbreak suggested even higher doses of GC to be effective among patients under critical conditions.^39^ While medium to high dose of GC can effectively reduce mortality of inflammation-induced respiratory distress via anti-inflammatory mechanisms, the mechanical rationale of potential protective effect of low dose GC against SARS-CoV-2-induced deterioration of respiratory function is still lacking. Taken together, the risk associated with GC therapy should be carefully considered, especially when treating patients with immunological disorders, and further analysis of the dose-dependent effect of GC is urgently needed to optimize the dose for different stages of COVID-19.

Despite of our findings of an IFN-GC synergy, the disruptive role of GC in IFN signaling is thought to interfere with IFN therapy in treating viral infections.^40^ We would attribute the strong synergy between IFN and GC despite of their functional conflicts partly to the use of inhaled IFN instead of intravenous or subcutaneous delivery methods in this cohort. The twice daily inhalation of IFN aerosol would deliver much higher concentration of IFN in lung epithelial cells than daily systemic GC, and the elicited IFN signaling might be sufficient to achieve clinical benefits even with the presence of GC. In fact, animal studies have shown that inhaled IFN could effectively repair the antiviral immunity impaired by GC and prevent viral infections.^16^ Another potential advantage of inhaled IFN is the low distribution of IFN to those air-impenetrable lung lesions where IFN may play a more damaging role in further exacerbating inflammation and disrupting tissue repair.^21,22^ Since GC is given systemically, they can still reach these regions via blood vessels to suppress inflammation. Taken together, we believe that the administration route of IFN may play a key role in achieving IFN-GC synergy in COVID-19,

Angiotensin-converting enzyme 2 (ACE2), the cell membrane adapter for SARS-CoV-2, was reported to be an interferon-stimulated gene (ISG), which raised potential risk of using IFN therapy to treat SARS-CoV-2 infection.^41^ However, latest evidence suggests that IFN induce an isoform of ACE2 not related to SARS-CoV-2 infection.^42,43^ Considering the complexity of IFN signaling and insufficient knowledge of COVID-19 immunopathogenesis, the therapeutic strategy of IFN should be carefully optimized in future trials. Also, it’s worth noting that since this study is retrospective, our definition of early IFN administration is only based on the characteristic of the studied cohort and may not be generalized. We would suggest that the time window for IFN therapy in clinical practice should be precisely determined based on the disease presentation instead of using fixed values due to the highly variable disease courses among severe patients.

Due to the retrospective design of this study, a number of factors with disease-modifying potential were different between the GC + IFN and GC only groups, which may interfere with the analysis. Although the initial assignment of IFN therapy was quasi-random, these differences in treatments suggests potential correlations between therapies, especially after the initial trial-and-error stage. For example, it is possible that the later use of GC in GC only group than IFN + GC group reflected the poorer disease prognosis without IFN that required GC therapy at later stages, while patients in IFN + GC group may have more severe manifestations at admission that warranted earlier GC use, but recovered faster, resulting in similar length and total dosage of GC therapy (Table 1). The discrepancy between antiviral treatments may also reflected the poor response to antiviral therapies other than IFN. While we do acknowledge these variations may affect the prognosis of disease, we believe that they are part of the standard care for emerging diseases and only randomized trials can properly address these variations.

Two potential mechanisms contributed to the paradoxical result of higher PVS prevalence among IFN only group despite of the antiviral potential of IFN. First, the negative effect of IFN on the integrity of lung epithelial may undermine the repairing process in the lung and impair viral clearance.^21,22^ While this effect may not significantly delay the symptomatic relief, it could extend the viral shedding period and lead to the increased prevalence of PVS. Another possibility is that administration of IFN via a nebulizer often induces temporary coughing, which may increase the viral load in the upper respiratory tract. Since administration of IFN and throat swab sampling for virological tests were usually both done in the morning schedule, there could be an artificial increase of PVS incidences among IFN users caused by inhalation of aerosol. Recent findings of the host genomic integration of SARS-CoV-2 sequences further complicate the clinical interpretation of positive virological tests after symptom relief and indicate that PVS may not always reflect delayed viral clearance in certain situations.^44^

As a side note, the longer LOS of this cohort than those in recent reports from the US and Europe is likely a result of improved detection sensitivity to SARS-CoV-2. Most patients in this cohort were diagnosed using less sensitive detection kits,^45^ leading to mild infections being undetected and thus excluded from analyses. In fact, most of those clinically diagnosed COVID-19 patients in China, who presented typical symptoms and radiological findings of COVID-19 but were virologically negative, had much shorter hospitalization than those tested positive for SARS-CoV-2, with a median LOS of 6 days among patients we surveyed. Thus, our cohort and findings may not represent mild cases.

Other than the above-mentioned issues, this study has several limitations. First, the nonrandomized assignment of therapies limits the interpretation of our findings. In particular, GC were given as an adjuvant therapy to patients with more serious presentation of COVID-19, which made assessment of IFN-GC synergy in mortality impossible due to the lack of a control group. IFN therapy was reported to be used quasi-randomly, but the requirement of nebulizers may impact patient assignment. However, the relatively high prevalence of GC and IFN exposure partially offset selection bias which was common in observational studies of GC. Second, the LOS values may be affected by the changing discharge criteria during the early outbreak. According to records, 12 of the 387 patients in this cohort were discharged before the enforcement of national guidelines that outlined the discharge criteria on February 2nd, 2020. Nonetheless, since our data were from a single hospital, the LOS values would not suffer inter-institution variation. Third, the prevalence of PVS could be affected by the different sensitivities of SARS-CoV-2 detection kits. Various investigational kits were used in January 2020, until a CFDA-approved kit (Sansure Biotech) was chosen and used exclusively after February 5th. We estimated that less than 10% of patients were discharged based on test results of investigational kits. Fourth, cytokine profiles and improvement of inflammation were not available in this study, which limited the mechanical interpretation of findings. Last, the conclusions of this study were based on single-center experience and prone to suffer unknown confounders.

In conclusion, our findings provide clinical evidence for an IFN–GC therapeutic synergy in COVID-19 and calls for further research into IFN–GC as a combination therapy.

## Materials and methods

### Patients

This non-interventional retrospective observational study was approved by Medical Ethics Committee of Suizhou Zengdu Hospital as a secondary analysis of identifiable data originally collected for non-research purposes with a waiver of informed consent. All patient identifications were replaced by anonymous codes during abstraction as stipulated by the Declaration of Helsinki.

During our prior study under the same protocol,^23^ we obtained all 406 inpatient records of confirmed COVID-19 patients diagnosed during January 15 through March 31. COVID-19 diagnosis was confirmed by two consecutive positive results of quantitative PCR-based SARS-CoV-2 nucleic acids tests of throat or nasal swab samples. All positive SARS-CoV-2 tests were subsequently verified at the laboratory of local CDC to eliminate false positives. Records were initially abstracted between May 8 and 22, 2020 into a standardized digital form based on the US CDC COVID-19 abstraction form with modifications to adapt local data and underwent daily quality control checks.^23^ Incomplete records of those also treated at other hospitals were subjected to a second round of search and abstraction between June 20 and 30, 2020 using the same methods. Survivors were followed-up every two weeks according to local regulations and the date of last recorded follow-up was May 22, 2020.

The exclusion criteria are: (1) incomplete records, such as receiving unknown therapies at another hospital for at least 5 days before being admitted or missing key laboratory or radiological results due to transfers, (2) requiring treatment for other conditions unrelated to COVID-19 that extended hospital stay for at least 5 days, (3) total hospital stay less than 5 days, and (4) received no antiviral therapy for more than 2 days during hospitalization because of lack of symptoms, late confirmation of diagnosis, severe adverse effects, or successful treatment of disease using supportive care. A final sample of 387 was analyzed in this study.

Patient information was collected on COVID-19 diagnosis, patient demographics, prior diagnosis of hypertension or diabetes, prior high-risk exposure such as close contact to COVID-19 patients or visiting high-risk locations, initial vital signs and laboratory test results within 24 h of admission, CT images and reports, and temporary and long-term prescriptions to describe the cohort and as potential confounders.

### Exposure

Per the national guideline, GC were given intravenously to patients with deteriorating oxygen saturation, rapid progression of lung lesions in CT scan, or evidence of hyperactivated immune system such as high fever and high plasma C-reactive protein.^28^ Cumulative methylprednisolone-equivalent doses of GC equal to or higher than 40 mg were recorded. Details of GC therapy were listed in Table 1. IFN-α2b and other antiviral medications were quasi-randomly used to treat confirmed COVID-19 patients.^28^ IFN-α2b was given at a dose of 5 million units in 2 mL sterile water via a nebulizer twice a day.^28^ IFN-α2b administered within the first 5 days of hospitalization for at least 3 days were deemed early IFN therapy.

### Outcomes

The primary outcome was hospital discharge. Secondary outcomes included symptom relief and prolonged viral shedding. The discharge criteria were defined in the national guideline as (1) normal body temperature for at least 3 days, (2) significant improvement of respiratory symptoms and function, (3) chest radiological imaging showing improvement of acute inflammation, (4) two consecutive negative SARS-CoV-2 nucleic acids tests of throat or nasal swab samples with at least 24 h interval between the two tests.^28^ Symptom relief was defined as normal body temperature for at least 3 days and significant improvement of respiratory symptoms and function.^28^ In severe and critical cases with disease relapse, symptom relief occurred before relapse was not counted. Prolonged viral shedding was defined as at least one positive SARS-CoV-2 nucleic acids test of throat or nasal swab samples more than 2 days after symptom relief. Due to limited testing capacity during the early outbreak, SARS-CoV-2 PCR tests were not conducted between confirmation of COVID-19 diagnosis and symptom relief.

### Sample size

An initial target sample size of 250 was determined based on the assumption of equal distribution of COVID-19 patients into the four treatment groups and α = 0.05. This sample size was calculated to have 90% power to detect a 15% increase of length of hospital stay (LOS) if the average LOS was 20, a previously observed value in Hubei.^23^ Sample size was calculated by G*power software (version 3.1).

### Statistical analysis

The distribution of treatment groups was summarized, and patient characteristics were assessed with Fisher’s exact test (dichotomous variables) or Chi-square test (variables with more than 2 categories) for categorical variables and Mann-Whitney U test for continuous variables. Cumulative incidence curves were compared by Log-rank test. Crude hazard ratios were calculated by logrank method. Adjusted hazard ratios were estimated by Cox proportional hazards models after adjusting for comorbidities as indicated in footnotes or legends. Proportional hazard assumption was tested by examination of the Kaplan-Meier curve. Crude odds ratios were calculated by Fisher’s exact test. Adjusted odds ratios were estimated by logistic regression after adjusting for comorbidities as indicated in footnotes or legends. Analyses were performed using SPSS 26 (IBM) or Prism 8 (GraphPad). Missing data were excluded pairwise from analyses. Significance was evaluated at α = 0.05 and all tests were 2-sided.

### Reporting summary

Further information on experimental design is available in the Nature Research Reporting Summary linked to this paper.

## Supplementary information

Supplementary materials

Reporting summary

## Data Availability

Correspondence and reasonable requests for original dataset should be addressed to Dr. Peng Hong (peng.hong@downstate.edu).
