# Supplementary material for: Clinical evidence of an interferon–glucocorticoid therapeutic synergy in COVID-19
Source: Signal Transduct Target Ther. 2021 Mar 3;6:107. doi: 10.1038/s41392-021-00496-5 (PMC7925812; doi:10.1038/s41392-021-00496-5)
Supplement: Supplementary file 1 — Supplementary materials [file 41392_2021_496_MOESM1_ESM.docx]

Supplementary Materials for

Clinical evidence of an interferon–glucocorticoid therapeutic synergy in COVID-19

Yingying Lu, Feng Liu, Gangling Tong, Feng Qiu, Pinhong Song, Xiaolin Wang, Xiafei Zou, Deyun Wan, Miao Cui, Yunsheng Xu, Zhihua Zheng, Peng Hong

Correspondence to: Dr. Peng Hong, [peng.hong@downstate.edu](mailto:peng.hong@downstate.edu)

**This PDF file includes:**

Figures. S1 to S6

Figure. S1.


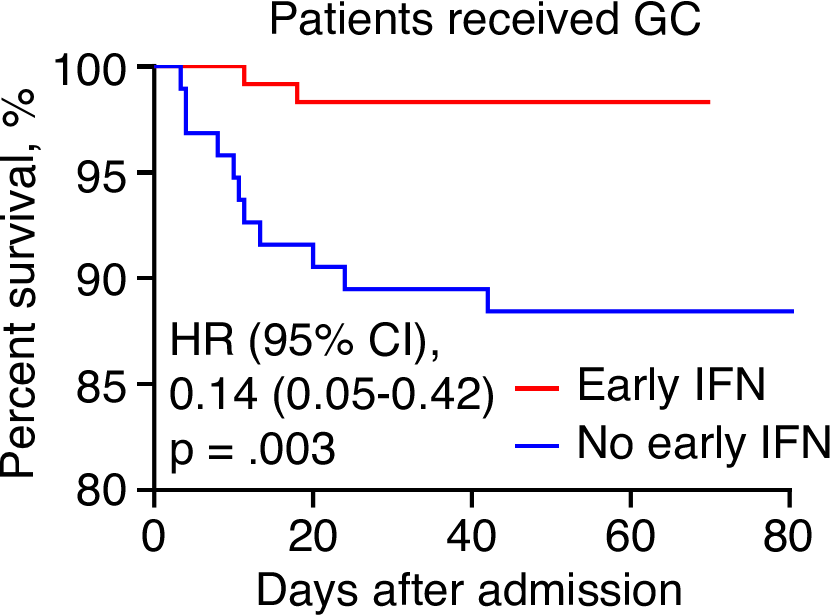


**In-hospital mortality among COVID-19 patients with GC.**

Kaplan-Meier curve of mortality in patients receiving GC during hospitalization. n = 118 (early IFN) and 98 (no early IFN).

Figure. S2.


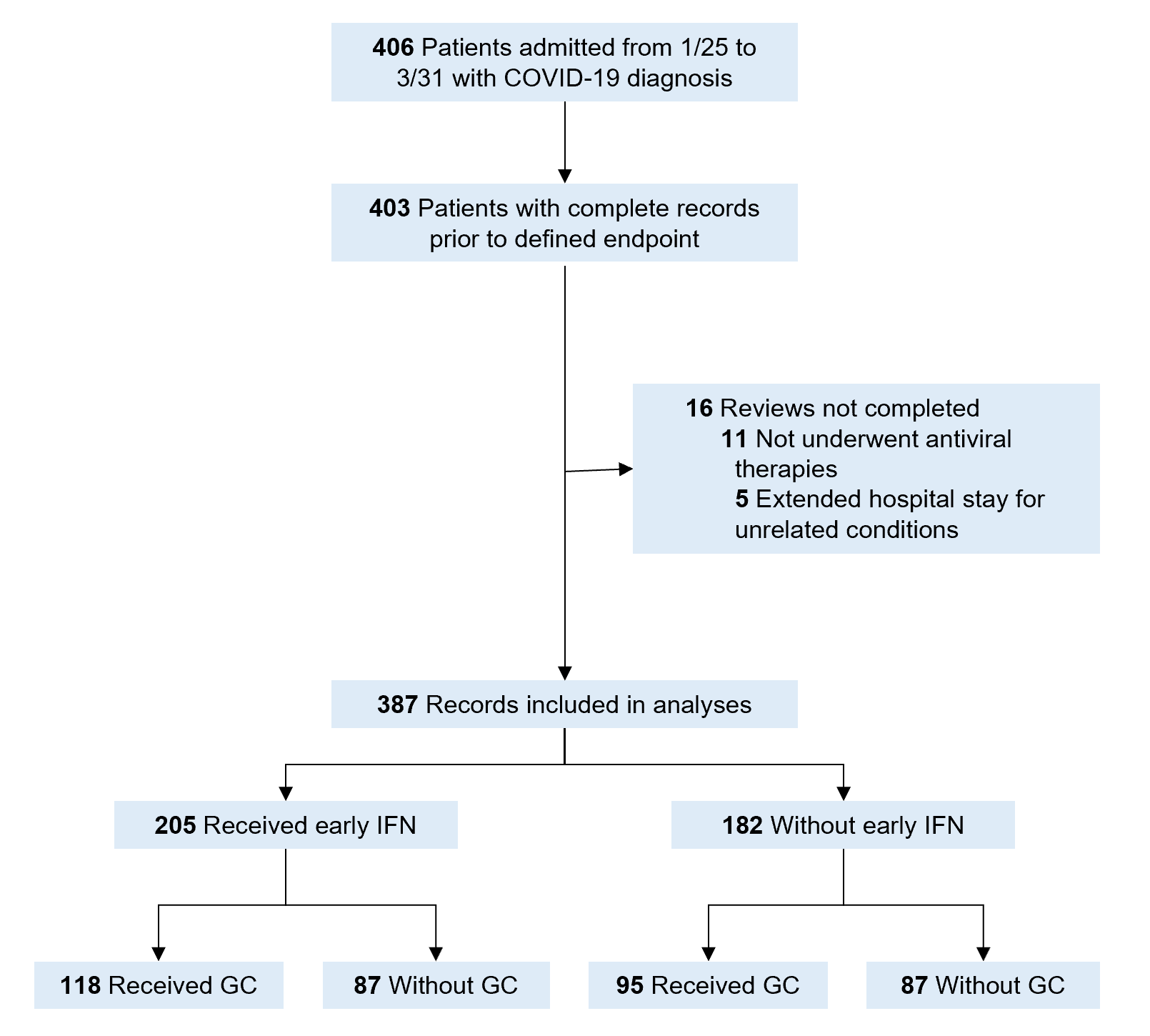
 **Sampling strategy of COVID-19 patient records.**

Figure. S3.

**
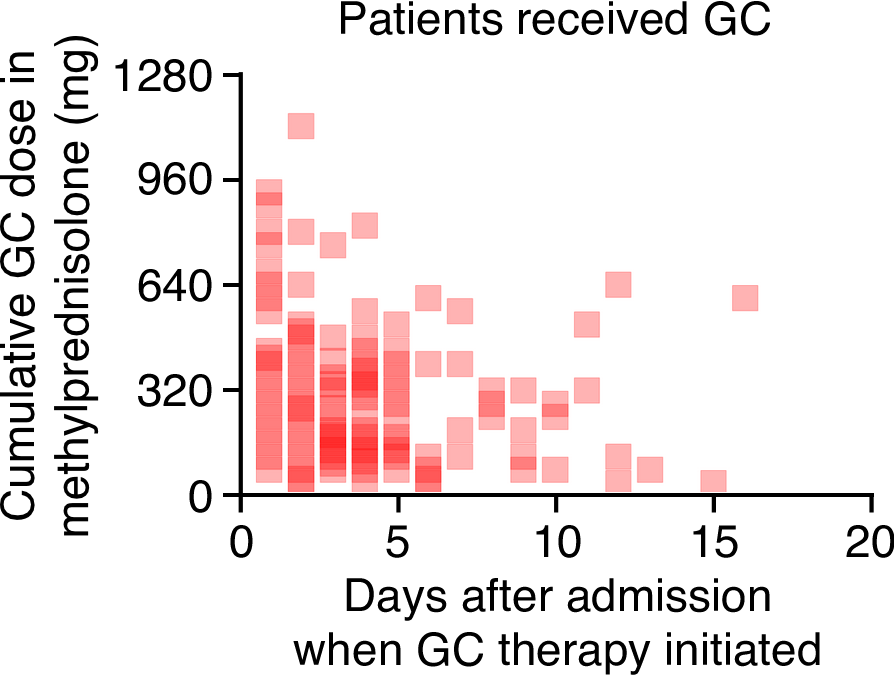
**

**Dot plot of the timing and cumulative doses of GC used in this cohort.**

n = 213.

Figure. S4.

**
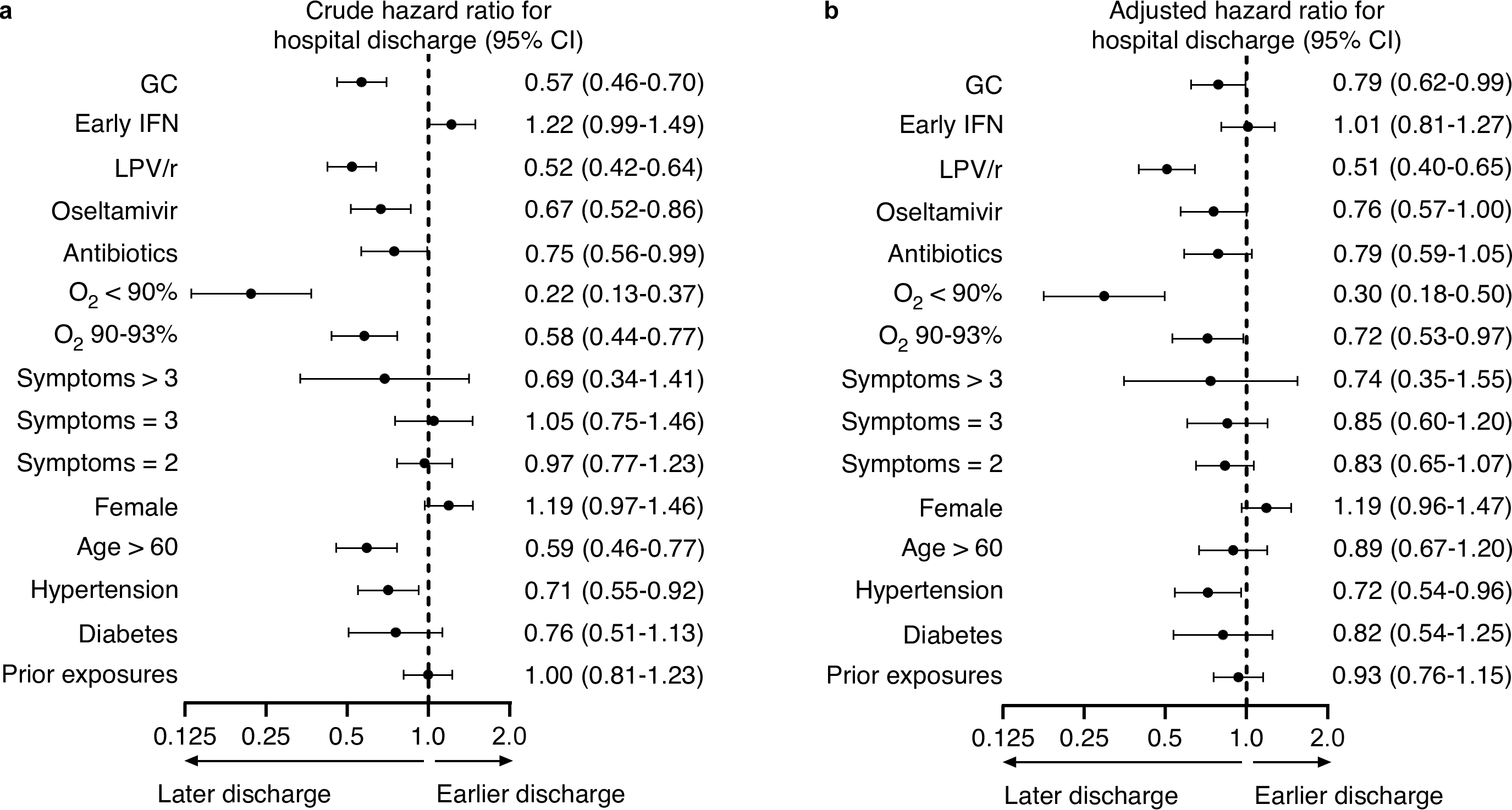
**

**Forest plots of HR of GC and confounders for hospital discharge.**

(**a**) Crude hazard ratios calculated by univariable Cox regression with only the indicated variable entered. All survivors were analyzed. n = 374. (**b**) Adjusted hazard ratios calculated by multivariable Cox regression with all listed variables entered. All survivors were analyzed. n = 374.

Figure. S5.

**
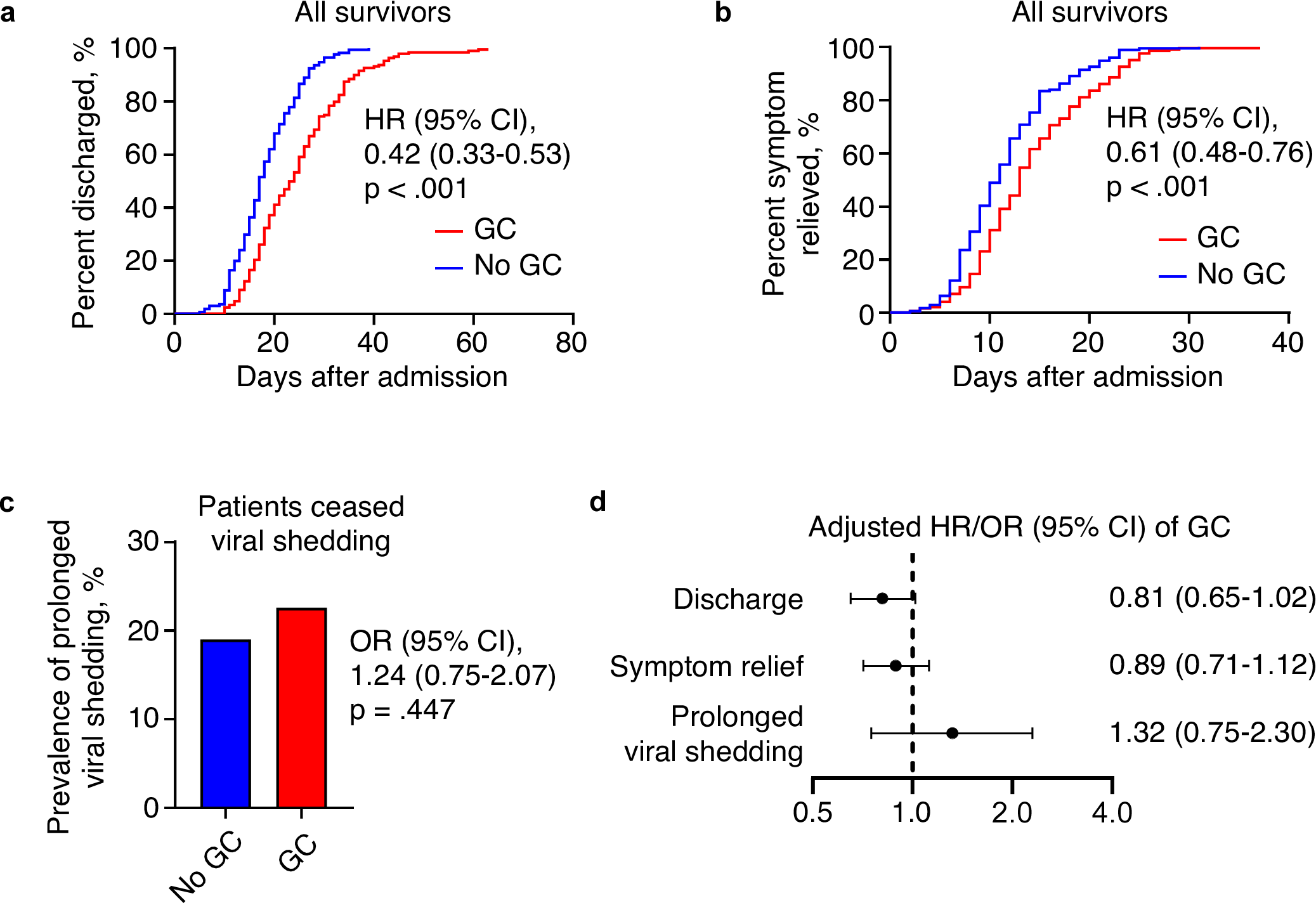
**

**Assessment of GC therapy on COVID-19 recovery.**

(**a,b**) Cumulative incidence curves for hospital discharge (a) and symptom relief (b). All survivors were analyzed. n = 200 (GC) and 174 (no GC). (**c**) Bar graph of prevalence rates of prolonged viral shedding. Patients ceased viral shedding before discharge or death were analyzed. n = 204 (GC) and 174 (no GC). (**d**) Adjusted HR and OR of GC for the indicated dependent variable. The statistical models were adjusted for gender, age, hypertension, diabetes, oxygen saturation at admission, LPV/r, oseltamivir, and antibiotics use. Cox models were fitted to all survivors (n = 374), and logistic regression were fitted to patients ceased viral shedding before discharge or death (n = 378).

Figure. S6.

**
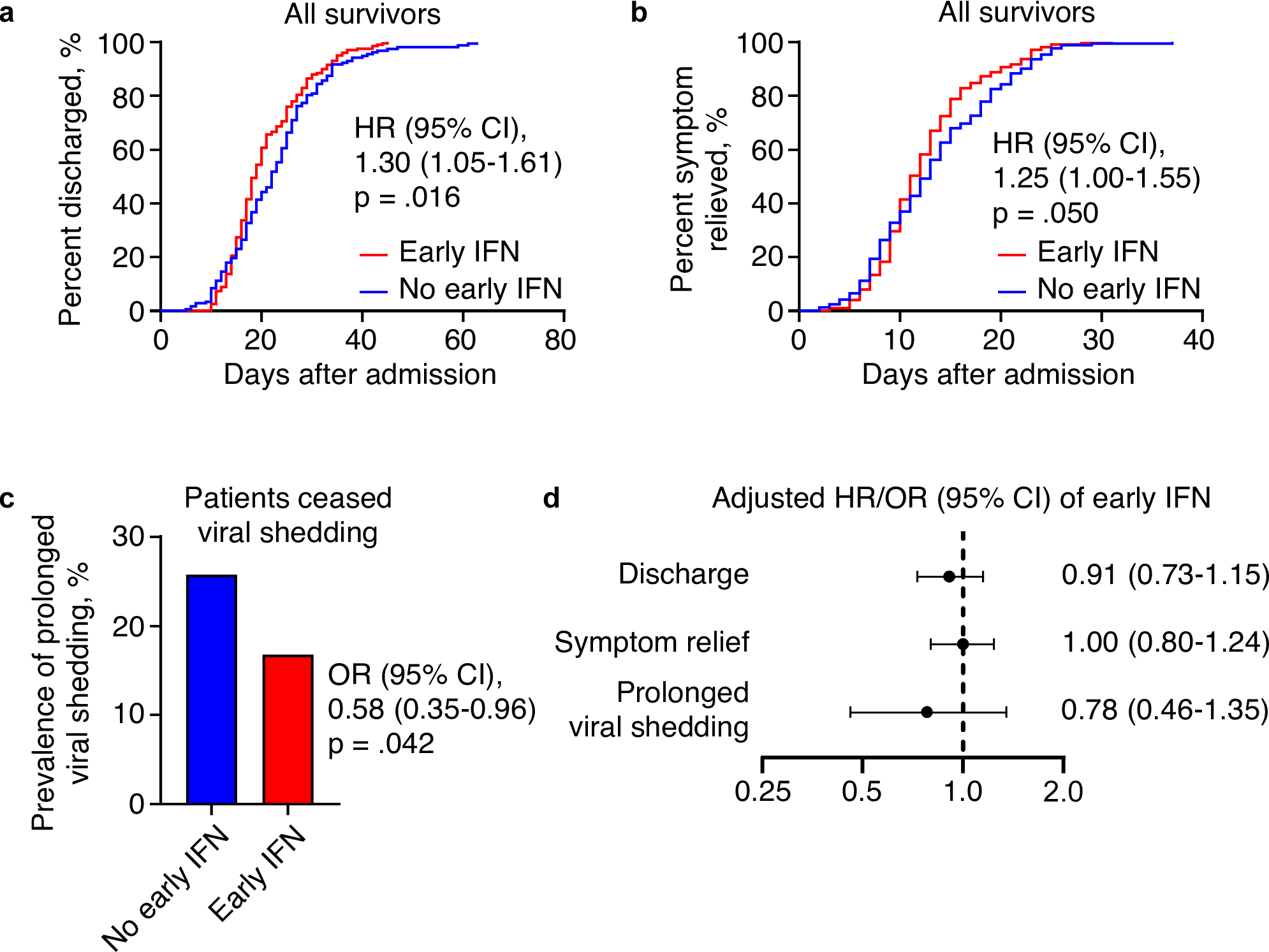
**

**Assessment of early IFN therapy on COVID-19 recovery.**

(**a,b**) Cumulative incidence curves for hospital discharge (a) and symptom relief (b). All survivors were analyzed. n = 203 (early IFN) and 171 (no early IFN). (**c**) Bar graph of prevalence rates of prolonged viral shedding. Patients ceased viral shedding before discharge or death were analyzed. n = 203 (early IFN) and 175 (no early IFN). (**d**) Adjusted HR and OR of early IFN for the indicated dependent variable. The statistical models were adjusted for gender, age, hypertension, diabetes, oxygen saturation at admission, LPV/r, oseltamivir, and antibiotics use. Cox models were fitted to all survivors (n = 374), and logistic regression were fitted to patients ceased viral shedding before discharge or death (n = 378).
